# Endothelial Glycocalyx in Kidney Transplantation: Molecular Mechanisms, Biomarkers, and Therapeutic Opportunities

**DOI:** 10.3390/ijms27104332

**Published:** 2026-05-13

**Authors:** Pavel Navratil, Minh Nguyet Tranova, Adam Haluska, Michal Lesko, Igor Gunka, David Astapenko

**Affiliations:** 1 Department of Urology, University Hospital Hradec Kralove, 500 05 Hradec Kralove, Czech Republic; 2 Faculty of Medicine in Hradec Kralove, Charles University, 500 03 Hradec Kralove, Czech Republicigor.gunka@fnhk.cz (I.G.);; 3 Department of Surgery, University Hospital Hradec Kralove, 500 05 Hradec Kralove, Czech Republic; 4 Department of Anesthesiology and Intensive Care Medicine, University Hospital Hradec Kralove, 500 05 Hradec Kralove, Czech Republic; 5 Faculty of Health Studies, Technical University in Liberec, 461 17 Liberec, Czech Republic; 6 Military Faculty of Medicine, University of Defence, 500 01 Hradec Kralove, Czech Republic

**Keywords:** endothelial glycocalyx, kidney transplantation, ischemia–reperfusion injury, syndecan-1, heparanase, angiopoietin-2, thrombomodulin, machine perfusion, extracellular vesicles, miR-126

## Abstract

The endothelial glycocalyx (EG) is a dynamic endothelial surface layer composed of proteoglycans, glycosaminoglycans, glycoproteins, and adsorbed plasma proteins that regulates permeability, mechanotransduction, leukocyte trafficking, coagulation, and nitric oxide signaling. In kidney transplantation (KT), the EG is exposed to cumulative injury from recipient uremia, donor instability, preservation, machine perfusion, reperfusion, rejection, and immunosuppressive toxicity. This narrative review summarizes EG biology in KT, with emphasis on biomolecular findings relevant to ischemia–reperfusion injury, delayed graft function, rejection, and chronic allograft injury. Particular attention is given to syndecan-1, heparan sulfate, heparanase, soluble thrombomodulin, matrix metalloproteinases, angiopoietin-2/Tie2 signaling, selectins, miR-126, extracellular vesicles, and urinary or perfusate-derived readouts. Current evidence is biologically coherent but uneven: human data are largely observational, whereas many therapeutic concepts remain preclinical or exploratory. Glycocalyx-centered phenotyping may eventually improve risk stratification and trial enrichment, but clinical implementation will require standardized sampling, sample-source-aware biomarker panels, prospective validation, and clear separation between mechanistic plausibility and proven clinical utility.

## 1. Introduction

Kidney transplantation (KT) remains the preferred treatment for end-stage kidney disease, but delayed graft function (DGF), rejection, and chronic allograft injury continue to limit long-term outcomes [1,2,3,4,5,6,7]. Current monitoring relies on serum creatinine, estimated glomerular filtration rate (eGFR), proteinuria, and functional trends, which are clinically useful but relatively late and nonspecific readouts. Earlier detection of endothelial and microcirculatory stress is therefore needed before graft dysfunction becomes clinically apparent.

The endothelial glycocalyx (EG), a luminal endothelial surface layer, may represent a common site where donor injury, cold ischemia, machine perfusion, reperfusion, alloimmune activation, and recipient uremic endotheliopathy converge [8,9,10,11,12,13]. However, the transplant literature remains fragmented: individual studies report soluble shedding markers, urinary fragments, perfusate signals, imaging readouts, or endothelial pathways, but these findings have rarely been integrated across donor-derived, graft-derived, recipient systemic, and post-transplant signals.

This review is therefore motivated by four unresolved questions: Which EG-related molecules are most relevant to KT? Which sample sources and time points are biologically interpretable? How strongly are current findings supported by human versus experimental evidence? What research designs are needed before glycocalyx-centered biomarkers or interventions can be translated into clinical decision-making?

## 2. Scope of This Review

This manuscript is a narrative review focused on mechanistic, translational, and clinically oriented literature relevant to EG biology in KT. Publications were selected through iterative searches of PubMed/MEDLINE, Scopus, and Google Scholar, last updated on 28 April 2026. Search strings combined endothelial glycocalyx terms (“endothelial glycocalyx”, “syndecan-1”, “heparan sulfate”, “heparanase”, “thrombomodulin”, “angiopoietin-2”, “Tie2”, “matrix metalloproteinase”, “selectin”), transplant-specific terms (“kidney transplantation”, “renal transplantation”, “ischemia–reperfusion injury”, “delayed graft function”, “machine perfusion”, “rejection”, “chronic allograft injury”), and emerging molecular readouts (“miR-126”, “microRNA”, “extracellular vesicles”, “exosomes”, “urinary extracellular vesicles”).

Eligible publications included original human KT studies, experimental kidney transplant or renal IRI models, organ-preservation studies, and mechanistic renal or CKD studies when they clarified biomarker behavior, endothelial biology, or therapeutic rationale applicable to KT. Foundational EG physiology papers were included when needed to explain molecular mechanisms. We excluded studies focused on unrelated vascular beds without a plausible renal or transplant link, nonrenal solid-organ transplantation without transferable endothelial mechanisms, conference abstracts without sufficient methodological detail, and reports describing nonspecific associations without relevance to EG biology.

Additional studies were identified by backward and forward citation tracking of key reviews and primary papers. No formal date restriction was applied, but more recent transplant, organ-preservation, and CKD biomarker studies were prioritized when multiple sources addressed the same question. Because the aim was a critical narrative synthesis rather than a systematic review, no PRISMA flow diagram, risk-of-bias assessment, or meta-analysis was performed. Throughout the review, we distinguish direct human KT evidence from experimental, ex vivo, and mechanistic evidence where this distinction affects clinical interpretation.

## 3. Molecular Architecture and Physiologic Role of the Endothelial Glycocalyx

The EG is a highly organized, dynamic endothelial surface layer rather than a uniform gel. Its core scaffold is formed by transmembrane proteoglycans, particularly syndecans and glypicans, which carry glycosaminoglycan side chains, mainly heparan sulfate, chondroitin sulfate, and, through interactions with membrane receptors and extracellular matrix components, hyaluronan [8,9,10]. These structural elements create a negatively charged, hydrated interface capable of binding albumin, antithrombin, growth factors, chemokines, extracellular superoxide dismutase, and other circulating mediators that collectively shape endothelial behavior [8,9,10].

Several functions of the EG are directly relevant to KT. First, it serves as a permeability barrier by limiting access of circulating macromolecules and cells to the endothelial membrane. Second, it acts as a mechanotransducer, whereby luminal shear stress is sensed through glycocalyx-linked signaling complexes that modulate endothelial nitric oxide synthase activity, nitric oxide release, and vascular tone. Third, it contributes to an anti-inflammatory and antithrombotic endothelial phenotype by spatially shielding adhesion molecules, preserving thrombomodulin- and heparan sulfate-dependent anticoagulant signaling, and dampening excessive leukocyte and platelet interactions [8,9,10,13].

In the kidney, these functions are especially important because glomerular and peritubular capillaries must maintain high-flow microvascular homeostasis while simultaneously protecting delicate filtration and tubular oxygen-delivery processes. The EG contributes to the charge- and size-selective properties of the glomerular filtration barrier and to the stability of the post-glomerular capillary network [14,15,16]. Accordingly, even subtle glycocalyx damage can have outsized physiologic consequences: increased permeability, altered shear responsiveness, endothelial swelling, leukocyte recruitment, and progressive capillary rarefaction.

Importantly, the EG is already abnormal in advanced kidney disease. Noninvasive and circulating biomarker studies show that worsening renal function is associated with thinning of the endothelial surface layer, elevated syndecan-1 and soluble thrombomodulin, higher angiopoietin-2, and broader evidence of endotheliopathy [14,17,18]. This matters in transplantation because the recipient endothelium is not reset to normality at the time of surgery; instead, KT takes place on a background of chronic uremic vascular stress, which likely modifies how the graft and the host respond to peri-transplant injury.

## 4. Mechanisms of Glycocalyx Injury Across the Kidney Transplant Continuum

A useful conceptual advantage of the EG is that it connects events occurring before implantation with those observed during early graft dysfunction and later chronic remodeling. Rather than treating KT injury as a single reperfusion event, a glycocalyx-centered framework views injury as cumulative, affecting both the graft and the recipient circulation.

### 4.1. Recipient Uremia, Dialysis Exposure, and Donor-Related Endothelial Priming

End-stage kidney disease promotes systemic endothelial dysfunction through oxidative stress, chronic inflammation, retained uremic solutes, altered shear conditions, and mineral–bone and metabolic abnormalities [7,14,17]. In this setting, the EG becomes thinner and more permeable, and circulating markers of glycocalyx damage rise. Liew and colleagues linked CKD-associated glycocalyx injury to uraemic toxins and showed that damage markers improve after successful KT, supporting the concept that part of the pre-transplant endothelial phenotype is driven by reversible metabolic stress [17,18].

Donor biology adds another layer of endothelial priming. Although direct human data remain limited, donor hemodynamic instability, catecholamine exposure, inflammation after brain death, and warm ischemic injury in circulatory-death donation are all likely to affect the graft endothelium before implantation [4,6,11,12]. In a deceased-donor cohort, serum syndecan-1 correlated with donor creatinine before procurement, suggesting that glycocalyx-related injury markers may reflect organ quality at the donor stage itself [19]. Even though recipient outcomes were not clearly predicted in that pilot study, the result is conceptually important: the graft may already carry a measurable glycocalyx injury signature before cold storage begins.

### 4.2. Cold Ischemia, Machine Perfusion, and Reperfusion-Triggered Shedding

Cold ischemia causes ATP depletion, endothelial swelling, cytoskeletal disorganization, and impaired ion homeostasis. Reperfusion then superimposes reactive oxygen species, calcium dysregulation, mitochondrial stress, leukocyte recruitment, complement activation, and coagulation pathway activation, all of which promote enzymatic shedding of glycocalyx components [4,11,12,13,20]. Human vascular surgery and multiple organ IRI models have shown that syndecan-1 and heparan sulfate are released into the circulation during ischemia–reperfusion, supporting glycocalyx shedding as an early response rather than a late epiphenomenon [13].

At the molecular level, several mechanisms converge on EG degradation. Matrix metalloproteinases (MMPs), heparanase, hyaluronidases, and related sheddases cleave structural components of the glycocalyx, while reactive oxygen species alter both the glycocalyx itself and endothelial signaling needed for its maintenance [11,13,16,21]. The kidney appears particularly vulnerable because the post-ischemic microcirculation can become trapped in a vicious cycle of glycocalyx loss, endothelial activation, leukocyte adhesion, capillary obstruction, and persistent local hypoxia.

Machine perfusion adds both opportunity and risk. On the one hand, oxygenated hypothermic or normothermic perfusion may improve energy repletion, allow graft assessment, and provide a platform for targeted endothelial protection. On the other hand, perfusion itself does not guarantee glycocalyx preservation. In porcine kidneys, normothermic machine perfusion was associated with measurable loss of endothelial glycocalyx regardless of tested pressure and hematocrit conditions, indicating that currently used perfusion settings may not be inherently glycocalyx-sparing [22]. This is highly relevant for KT because perfusate composition, flow characteristics, colloid support, red cell handling, and inflammatory burden may become modifiable determinants of graft endothelial biology during ex situ preservation [12,22].

### 4.3. Complement, Coagulation, and Leukocyte Trafficking After Glycocalyx Disruption

The EG normally functions as a spatial and biochemical shield for the endothelial cell. Once the layer is degraded, adhesion molecules and procoagulant surfaces become more accessible, and the endothelium shifts from a quiescent to an activated phenotype [8,9,10,11,12,13]. Complement-mediated injury is one important amplifier of this transition. In experimental renal IRI, complement has been shown to damage the glycocalyx directly, thereby exacerbating vascular permeability and inflammatory injury [20]. The relationship is bidirectional: complement activation damages the glycocalyx, while glycocalyx loss further facilitates inflammatory amplification.

The coagulation axis is also deeply intertwined with EG integrity. Heparan sulfate side chains and thrombomodulin-dependent signaling contribute to the anticoagulant endothelial phenotype. When glycocalyx structure is lost, endothelial thrombomodulin shedding and reduced protein C activation can promote a local prothrombotic milieu [23,24]. The same process likely contributes to post-transplant microvascular dysfunction and the “no-reflow” component of DGF. In parallel, exposed adhesion receptors favor leukocyte rolling, firm adhesion, and transendothelial migration, supporting both innate inflammatory injury and alloimmune processes [6,7,11].

An additional molecular insight comes from complement factor H. The EG, particularly its heparan sulfate-rich domains, contributes to factor H binding. Cyclosporine-induced endothelial injury has been shown to impair this interaction, thereby linking drug toxicity, glycocalyx loss, and susceptibility to complement-mediated endothelial damage [25]. This observation is particularly relevant for KT because it connects classic transplant exposures—calcineurin inhibition, complement activation, and endothelial dysfunction—through a shared glycocalyx-dependent mechanism.

### 4.4. Rejection, Drug Toxicity, and Chronic Microvascular Remodeling

The molecular relevance of the EG likely extends beyond early IRI. Endothelial injury is central to both T-cell-mediated and antibody-mediated graft damage, and the microvasculature is a major target of chronic allograft deterioration [6,7]. Although the transplant literature is still evolving, several molecular signals suggest that glycocalyx injury participates in this broader continuum. Plasma heparan sulfate has been reported to rise in kidney transplant recipients with biopsy-proven acute cellular rejection, consistent with heparanase-driven matrix and glycocalyx degradation during alloimmune cell infiltration [26].

In antibody-mediated rejection, extracellular matrix turnover and endothelial activation are strongly intertwined. A recent IJMS paper from this Special Issue demonstrated altered plasma profiles of MMP-1, MMP-2, MMP-3, and TIMP-3 in kidney transplant recipients with acute antibody-mediated rejection, reinforcing the relevance of protease-regulated endothelial and matrix remodeling in graft injury [27]. While MMP/TIMP measurements are not glycocalyx-specific, they mechanistically align with syndecan and heparan sulfate shedding biology and may form part of a broader endothelial injury panel.

Chronic remodeling is likely driven by repeated or persistent endothelial perturbation rather than a single event. Heparanase has been linked to chronic kidney dysfunction and fibrosis after IRI, suggesting that early glycocalyx injury can propagate long-term maladaptive repair [21]. Likewise, endothelial dysfunction in KT has been associated with later cardiovascular and renal risk, meaning that glycocalyx damage may influence both graft survival and the broader vascular health of the recipient [6,7]. In this sense, the EG may be viewed as both a biomarker source and a mechanistic hub that connects acute injury to chronic microvascular attrition.

## 5. Biomolecular Findings and Biomarkers in Kidney Transplantation

Translational interest in the EG is driven by the possibility that molecular signatures of endothelial injury can be measured in clinically accessible sample sources. However, not all biomarkers report the same biology. Some reflect structural shedding, others endothelial activation, and others broader vascular destabilization or matrix turnover. Their interpretation therefore depends on timing, sample source, and clinical context (Table 1).

### 5.1. Syndecan-1: The Most Studied Glycocalyx Shedding Marker

Syndecan-1 is the most widely used circulating marker of glycocalyx injury. It is a transmembrane heparan sulfate proteoglycan whose ectodomain is shed during endothelial stress. In CKD, syndecan-1 rises with worsening uremic burden and correlates with other features of endothelial dysfunction [14,17]. After successful KT, biochemical glycocalyx markers, including syndecan-1, improved over the subsequent months, indicating at least partial reversibility of the uremia-related endothelial phenotype [18].

Beyond its practical role as a soluble shedding marker, syndecan-1 is mechanistically relevant to CKD pathogenesis. As a heparan sulfate proteoglycan, it organizes the endothelial surface layer, contributes to ligand binding and chemokine gradients, and helps maintain the barrier, anti-adhesive, and antithrombotic properties of the microvascular interface. CKD-associated uremic toxins, inflammation, and oxidative stress can accelerate ectodomain shedding, thereby converting syndecan-1 from a structural component of endothelial protection into a circulating signal of endotheliopathy [17,32]. This is particularly important in KT because the recipient arrives for transplantation with a pre-existing systemic endothelial injury phenotype rather than a normal vascular baseline.

The interaction between syndecan-1 and miR-126 adds an additional regulatory dimension. miR-126 is an endothelial-enriched microRNA involved in vascular homeostasis and endothelial repair. In a large CKD cohort across disease stages, Fourdinier et al. showed that circulating miR-126 was associated with syndecan-1 and selected uremic toxins, particularly free indoxyl sulfate and total p-cresyl glucuronide, in multivariable analyses [32]. This supports a model in which CKD-related endotheliopathy is not limited to passive glycocalyx shedding but also involves altered endothelial microRNA signaling. For transplantation, the syndecan-1/miR-126 axis may therefore help connect recipient uremic priming, endothelial vulnerability at reperfusion, and post-transplant recovery of vascular homeostasis.

Within transplantation, the promise of syndecan-1 lies in its biologic plausibility across several sample sources. Donor serum syndecan-1 correlates with donor kidney function before procurement [19]. Experimental KT work from the authors’ group showed that syndecan-1 decreases early after sulodexide treatment in a porcine reperfusion model, supporting its responsiveness to glycocalyx-directed intervention [30]. In addition, non-transplant perioperative studies demonstrate that postoperative syndecan-1 is associated with AKI risk, strengthening the notion that syndecan-1 captures clinically meaningful endothelial injury rather than a purely descriptive molecular event [31].

That said, syndecan-1 is not perfectly specific. It can be influenced by systemic inflammation, fluid shifts, surgery, and nonrenal vascular injury. Its clinical utility in KT will therefore likely depend on serial sampling, integration with other markers, and interpretation within defined windows such as donor assessment, reperfusion, DGF evaluation, or rejection work-up. The central translational question is not whether syndecan-1 is “the” biomarker, but whether dynamic syndecan-1 behavior can identify a reproducible endothelial injury phenotype within KT.

### 5.2. Soluble Thrombomodulin and the Endothelial Anticoagulant Axis

Thrombomodulin is an endothelial membrane glycoprotein with a key role in protein C activation and maintenance of an antithrombotic endothelial state. Once shed, soluble thrombomodulin (sTM) serves as a marker of endothelial cell injury rather than structural glycocalyx loss alone. This difference matters: sTM may be particularly informative when the insult extends beyond glycocalyx cleavage to overt endothelial membrane perturbation.

Several observations support the relevance of sTM in KT. In the CKD-to-transplant continuum, sTM rises as renal function worsens and improves after transplantation alongside other endothelial markers [14,18]. A recent transplant study comparing simple hypothermia with hypothermic machine perfusion found early reperfusion differences in renal vein sTM kinetics, suggesting that preservation strategy may influence the severity of immediate endothelial injury at the graft level [24]. Experimentally, soluble thrombomodulin also has protective properties in ischemic kidneys, indicating that the thrombomodulin–protein C axis is not only a biomarker pathway but also a potential therapeutic one [23].

The main limitation of sTM is interpretive breadth. It may capture severe endothelial injury but does not localize the lesion to the graft unless sampling is organ-specific. For this reason, future KT studies may benefit from paired systemic and graft-proximal sampling, particularly when evaluating preservation technologies or immediate reperfusion biology.

### 5.3. Heparan Sulfate, Heparanase, Hyaluronan, and Urinary Glycocalyx Fragments

Heparan sulfate is the dominant sulfated glycosaminoglycan within the endothelial glycocalyx and a major determinant of its charge properties and ligand interactions [8,9,10,16]. Its degradation is catalyzed by heparanase, an endoglucuronidase that has emerged as a mechanistically important amplifier of renal inflammation, protein leakage, and fibrosis [16,21]. In renal disease models, heparanase upregulation worsens injury, while heparanase inhibition protects against chronic dysfunction after IRI [21]. This makes the heparan sulfate/heparanase axis particularly attractive in KT, where early endothelial and basement-membrane injury may shape long-term repair.

In human transplantation, plasma heparan sulfate has been associated with biopsy-proven acute cellular rejection, supporting its use as a noninvasive readout of alloimmune endothelial–matrix injury [26]. In the experimental KT setting, urinary glycosaminoglycans have been used as a complementary measure of glycocalyx disruption and were reduced by albumin treatment in a porcine model, suggesting that urine may provide a graft-proximal, noninvasive window into glycocalyx damage [30].

Compared with syndecan-1, heparan sulfate and urinary glycosaminoglycan measurements may offer closer biologic linkage to the structural glycosaminoglycan network itself. Their disadvantages are lower standardization, stronger dependence on assay methodology, and greater uncertainty about how much signal derives from endothelial versus epithelial, basement-membrane, or broader extracellular matrix turnover. These are not reasons to abandon them; rather, they argue for more explicit sample-source-aware study design.

### 5.4. Angiopoietin-2/Tie2 Signaling, VEGF Pathways, Selectins, and Matrix Remodeling

The Angiopoietin/Tie2 axis is one of the most attractive molecular frameworks connecting EG integrity with endothelial quiescence. Tie2 signaling stabilizes the endothelium, preserves barrier function, and suppresses inflammatory activation. In ischemic kidney injury, Tie2 activation protects renal function, while VE-PTP-driven Tie2 inhibition worsens endothelial vulnerability [33]. The translational implication for KT is substantial: Angiopoietin-2 may behave as a vascular destabilization biomarker, whereas Tie2-directed therapies may become an endothelial protection strategy [33,34].

Selectins and other adhesion molecules report endothelial and platelet activation, but their behavior in KT appears more variable than that of syndecan-1 or sTM. In a recent perioperative KT cohort, several endothelial injury markers changed dynamically after transplantation, yet the signal was heterogeneous and not all markers discriminated meaningful clinical subgroups [29]. This variability should not be read as failure. Rather, it suggests that endothelial activation markers may be highly dependent on donor type, immunosuppression, operative timing, and recipient systemic inflammation.

Matrix turnover markers may prove especially useful in immunologic injury. In antibody-mediated rejection, altered MMP and TIMP profiles have already been demonstrated in an IJMS article from the same Special Issue [27]. Because glycocalyx degradation and extracellular matrix remodeling share several proteolytic pathways, future biomarker panels may need to combine structural shedding markers (such as syndecan-1 or heparan sulfate) with pathway-activity markers (such as MMP/TIMP signatures, Ang-2, or endothelial growth factor-related signals) rather than relying on a single analyte.

### 5.5. Perfusate, Urinary, and Microvascular Readouts: Moving Beyond Plasma Alone

One of the main lessons from existing studies is that the biomarker sample source matters. Plasma markers are easy to obtain but are influenced by systemic recipient factors. Perfusate and early renal venous measurements may be more graft-specific and particularly useful for organ preservation research [12,22,24]. Urinary glycocalyx fragments may better reflect the allograft than peripheral blood and deserve more systematic study [30]. Noninvasive microvascular imaging adds a functional layer: the perfused boundary region, used as a surrogate of endothelial surface layer dimensions, improves after KT and may complement biochemical measurements [14,18].

The next step is not to choose one sample source over another, but to build linked datasets. For example, donor blood, perfusate, reperfusion venous samples, recipient plasma, urine, and histology could be integrated to separate donor-derived endothelial damage from recipient systemic endotheliopathy and to identify which signals are most predictive of DGF, rejection, or chronic dysfunction. A biomarker becomes clinically useful only when the sample source, the time window, and the decision context are all explicit.

### 5.6. Extracellular Vesicles: Linking Glycocalyx Injury, Alloimmunity, and Graft-Specific Readouts

Extracellular vesicles (EVs), including exosomes and microvesicles, are increasingly relevant to KT because they combine features of biomarkers and mediators. They carry proteins, lipids, mRNA, microRNAs, and surface antigens that reflect the cell of origin and the state of injury or activation. From a glycocalyx perspective, endothelial EVs may accompany EG disruption as part of endothelial activation, membrane remodeling, and coagulation-inflammatory signaling; their cargo could include glycoproteins, proteases, and regulatory RNAs that modulate barrier integrity. However, EVs are not simple replacements for soluble EG fragments: they report cell-derived vesicular communication, whereas syndecan-1 and heparan sulfate report structural shedding. Combined measurement may help identify whether endothelial injury is dominated by shedding, immune activation, tubular injury, or microvascular remodeling [35,36,37,38].

EVs are particularly relevant to rejection biology. They can carry donor major histocompatibility complex molecules and peptides and participate in alloantigen transfer, cross-dressing of recipient antigen-presenting cells, T-cell allorecognition, and immune regulation [36,37]. In a recent cohort including 92 CKD patients, 70 KT recipients, and 33 healthy volunteers, Jacob et al. quantified urine and plasma EVs and EV subpopulations. In CKD, total urinary EV concentration correlated positively with eGFR, but no EV subgroup clearly identified nephropathy; in KT recipients, antibody-mediated rejection was associated with lower plasma EVs derived from B cells, T cells, and endothelium, while T-cell-mediated rejection was associated with lower urinary endothelial EVs [35]. These findings suggest that EV size, concentration, sample source, and cellular origin may vary across CKD severity and rejection phenotypes.

For glycocalyx-centered KT research, EVs should therefore be interpreted as a complementary layer rather than a standalone diagnostic. Plasma endothelial EVs may reflect systemic endotheliopathy, while urinary or kidney-derived EVs may be more graft-proximal. Integrating EV phenotyping with syndecan-1, heparan sulfate, sTM, Ang-2/Tie2 signaling, MMP/TIMP profiles, urine markers, perfusate measurements, and biopsy or transcriptomic data could help distinguish donor-derived endothelial injury from recipient uremic endotheliopathy and active alloimmune injury.

## 6. Therapeutic Opportunities to Preserve or Restore the Glycocalyx: Evidence Levels and Limitations

**Figure 1:**
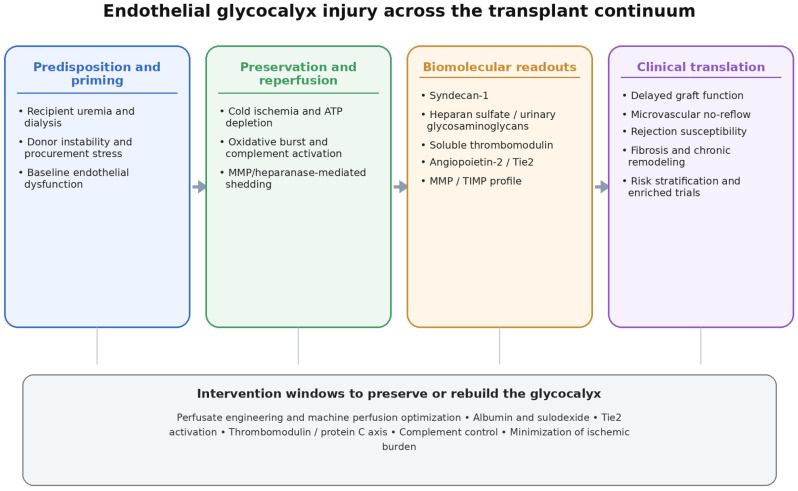
Conceptual overview of endothelial glycocalyx injury across the kidney transplant continuum and principal intervention windows. Abbreviations: ATP, adenosine triphosphate; MMP, matrix metalloproteinase; TIMP, tissue inhibitor of metalloproteinases; Tie2, TEK receptor tyrosine kinase (tyrosine kinase with immunoglobulin-like and EGF-like domains 2).

If the EG participates upstream in transplant injury, it is a rational therapeutic target; however, the supporting evidence is heterogeneous and should not be read as clinical readiness. At present, no glycocalyx-directed therapy is established as standard care in KT. Human evidence consists mainly of observational biomarker studies and graft-preservation comparisons, whereas interventional support is largely derived from kidney IRI models, ex vivo perfusion work, or small preclinical transplant models. The term “therapeutic opportunities” is therefore used here in a translational sense: these strategies are candidate approaches for mechanistic studies and trial design, not current treatment recommendations (Table 2 and Figure 1).

### 6.1. Preservation Strategy and Perfusate Engineering

Because significant endothelial injury develops before or at reperfusion, preservation is an obvious interventional window. Hypothermic and normothermic machine perfusion create an opportunity to manipulate perfusate composition, shear conditions, oxygen delivery, colloid support, and inflammatory burden before implantation [12,22]. However, the observation that normothermic perfusion itself can be accompanied by glycocalyx loss is a warning that “more physiologic” preservation is not automatically glycocalyx-protective [22]. Future perfusate design should therefore consider specific endothelial endpoints rather than relying only on global function markers or macroscopic perfusion parameters.

Potential perfusate goals include better control of oxidative stress, preservation of heparan sulfate-rich interfaces, prevention of endothelial swelling, reduction in hemolysis-related damage, and maintenance of the oncotic environment required for EG stability. Because the EG depends on both structural integrity and continuous molecular renewal, preservation solutions may need to be evaluated not only for what they prevent, but also for what they fail to replace.

### 6.2. Albumin, Sulodexide, and Glycosaminoglycan-Oriented Approaches

Albumin has a special place in glycocalyx biology because it contributes to the oncotic and electrostatic environment of the endothelial surface layer and may help preserve barrier properties [10,12]. Sulodexide, a glycosaminoglycan-based drug with endothelial and antithrombotic effects, is another attractive candidate because it may support glycocalyx rebuilding and modulate heparanase-dependent injury. In a porcine KT model, albumin reduced urinary glycosaminoglycan release and sulodexide lowered early serum syndecan-1 after reperfusion, providing proof-of-concept that glycocalyx-directed intervention can shift measurable injury signals during KT-like IRI [30].

These findings should be interpreted cautiously and should not be taken as evidence of clinical efficacy. The experimental effects were early, the sample size was small, and histologic differences were minor. Their value is primarily proof-of-concept: they show that glycocalyx-directed interventions can shift measurable injury signals during KT-like IRI. Larger preclinical studies and carefully designed human feasibility studies are needed before albumin, sulodexide, or related compounds can be considered therapeutic candidates in routine KT care.

### 6.3. Tie2 Activation, Thrombomodulin–Protein C, and Complement Control

Tie2 activation is one of the better-developed mechanistic endothelial protection strategies. In kidney IRI, genetic or pharmacologic reinforcement of Tie2 signaling protects the endothelium and improves renal outcomes [33]. Earlier transplant-focused work has proposed Tie2 modulation as a promising direction [34]. Nevertheless, its relevance to clinical KT remains inferential until timing, dosing, safety, and patient selection are tested in transplant-specific models and, ultimately, human studies.

The thrombomodulin–protein C axis and complement control are also plausible, but the same caution applies. Soluble thrombomodulin protects ischemic kidneys experimentally, and clinical transplant studies suggest that thrombomodulin kinetics reflect early graft endothelial injury [23,24]. Complement-mediated glycocalyx injury has been shown in renal IRI models [20], while complement activation is central to several forms of transplant injury. These observations support mechanistic prioritization, but they do not yet establish biomarker-guided treatment responsiveness in KT.

### 6.4. Perioperative and Post-Transplant Supportive Strategies

Not all glycocalyx-preserving strategies will be drug-based. Avoidance of unnecessary ischemic burden, minimization of endothelial-toxic exposures, careful control of hemodynamics, judicious fluid management, and early detection of endothelial stress may all contribute to preserving the microvascular interface after KT. In addition, the relationship between immunosuppressive toxicity and glycocalyx loss—illustrated by cyclosporine-related impairment of factor H binding—suggests that endothelial biology should be considered when evaluating maintenance immunosuppression and its vascular side effects [25].

Supportive care and targeted therapy may eventually be combined, but the current clinical implication is more conservative: avoid preventable endothelial stress while using glycocalyx-related measurements primarily as research tools. A glycocalyx-based approach is less about a single molecule and more about defining a vascular injury phenotype that can be reproducibly measured, linked to outcomes, and then tested in intervention studies.

## 7. Critical Gaps and Research Implications

The major limitation of the field is not the lack of biologic plausibility, but the lack of sample-source-specific validation. Plasma markers are easy to measure but are confounded by recipient systemic inflammation, dialysis exposure, surgery, fluid shifts, and nonrenal vascular injury. Urine and perfusate markers are more graft-proximal but require normalization, standardized collection, and correlation with biopsy or functional endpoints. Renal venous sampling is biologically attractive but logistically difficult and rarely available beyond the immediate reperfusion period.

A second gap is temporal resolution. Many studies use isolated perioperative or postoperative samples, although EG injury is likely dynamic and phase-specific. Donor injury, cold ischemia, reperfusion, DGF, rejection, and chronic remodeling may generate different marker patterns. Future studies should predefine sampling windows such as donor assessment, machine perfusion, pre-implantation, early renal venous reperfusion, postoperative days 1–7, and follow-up at 1–3 months, rather than pooling all signals into a generic category of endothelial injury.

Concrete research implications follow from these gaps. Prospective KT cohorts should combine syndecan-1, heparan sulfate or urinary glycosaminoglycans, sTM, Ang-2/Tie2-related markers, MMP/TIMP profiles, EV phenotyping, and miR-126 only when the study design can specify what each analyte is expected to represent. These panels should be paired with histology, transcriptomics or single-cell endothelial signatures, perfusion parameters, and prespecified outcomes such as DGF, biopsy-proven rejection, eGFR recovery, eGFR slope, proteinuria, fibrosis, and graft survival.

For clinical practice, the immediate implication is caution. EG-related markers are not ready as stand-alone diagnostics, routine surveillance tests, or triggers for treatment. Their near-term value is more likely in trial enrichment, pharmacodynamic monitoring, and mechanistic endotyping of high-risk settings such as donation after circulatory death, prolonged cold ischemia, expanded-criteria donation, severe recipient uremic endotheliopathy, or early DGF. Only after external validation, assay harmonization, and demonstration of incremental value beyond existing clinical variables should glycocalyx-centered phenotyping be considered for clinical algorithms.

## 8. Conclusions

The endothelial glycocalyx offers a coherent molecular framework for understanding endothelial injury in KT, from donor instability and preservation to reperfusion, rejection, and chronic microvascular remodeling. Its disruption is biologically plausible as a link between IRI, endothelial activation, impaired microcirculation, inflammation, thrombosis, and fibrosis. Among currently studied readouts, syndecan-1 is the most mature structural shedding marker, while soluble thrombomodulin, heparan sulfate/heparanase-related measures, Angiopoietin/Tie2 signals, MMP/TIMP profiles, miR-126, and EV-derived signatures provide complementary but less standardized information.

The evidence base supports cautious conclusions rather than immediate translation. First, EG injury in KT is measurable and mechanistically relevant, but interpretation depends strongly on sample source, timing, assay method, and whether the readout reflects structural shedding, vesicular communication, endothelial activation, or broader matrix turnover. Second, therapeutic targeting of the EG remains exploratory: supportive strategies such as ischemia minimization and optimized preservation are clinically sensible, whereas albumin, sulodexide, Tie2 modulation, thrombomodulin–protein C support, and complement-directed approaches require further preclinical and human validation. The next step is not routine clinical implementation but standardized, multimodal, prospective research that tests whether glycocalyx-centered phenotyping adds prognostic or therapeutic value beyond current transplant practice.

## Figures and Tables

**Table 1:** Molecular components and candidate biomarkers of the endothelial glycocalyx relevant to kidney transplantation.

Molecule/Axis	Biologic Meaning	Common Sample	Potential Relevance in KT
Syndecan-1	Ectodomain shedding of a transmembrane heparan sulfate proteoglycan; surrogate of structural glycocalyx injury	Plasma/serum	Donor organ quality assessment, peri-reperfusion endothelial injury, DGF-risk phenotyping, longitudinal endothelial recovery [17,18,19,28,29,30,31]
miR-126/syndecan-1 axis	Endothelial-enriched microRNA linked to vascular homeostasis; association with syndecan-1 and uremic toxins suggests integrated CKD endotheliopathy	Plasma/serum	Recipient endothelial priming, CKD-related endothelial vulnerability before KT, adjunct RNA-based biomarker alongside structural glycocalyx markers [32]
Heparan sulfate/urinary glycosaminoglycans	Direct readout of glycosaminoglycan degradation; closely linked to heparanase activity and endothelial–matrix injury	Plasma, urine, perfusate	Acute cellular rejection biology, graft-proximal injury monitoring, experimental evaluation of glycocalyx-preserving therapies [16,21,26,30]
Soluble thrombomodulin	Endothelial membrane injury and loss of anticoagulant surface signaling	Plasma/serum, renal venous blood	Early reperfusion injury, comparison of preservation strategies, complement/coagulation-linked endotheliopathy [14,18,23,24]
Angiopoietin-2/Tie2 pathway	Vascular destabilization versus endothelial quiescence signaling	Plasma, tissue, transcriptomics	Risk stratification in IRI, mechanistic target for endothelial protection, linkage to permeability and inflammation [14,33,34]
MMP/TIMP profile	Protease activity and matrix remodeling; may contribute to glycocalyx cleavage and endothelial remodeling	Plasma/serum, tissue	Antibody-mediated rejection, inflammatory matrix turnover, adjunct biomarker panel rather than standalone glycocalyx marker [13,27]
Selectins and adhesion molecules	Endothelial or platelet activation after loss of quiescent luminal shielding	Plasma/serum	Perioperative endothelial activation signal, but currently less specific and more variable than structural shedding markers [29]
Perfused boundary region (PBR)	Functional microvascular surrogate of endothelial surface layer dimensions	Sublingual intravital imaging	Noninvasive longitudinal endothelial phenotyping before and after KT; complementary to circulating markers [14,18]
Extracellular vesicles (EVs)	Cell-derived vesicular communication; size, concentration, cellular origin, and cargo reflect endothelial, immune, tubular, or graft-specific activation	Plasma, urine, perfusate (experimental)	Rejection phenotyping, graft-proximal urinary monitoring, separation of endothelial shedding from active intercellular signaling [35,36,37,38]

Abbreviations: DGF, delayed graft function; KT, kidney transplantation; MMP, matrix metalloproteinase; TIMP, tissue inhibitor of metalloproteinase.

**Table 2:** Candidate glycocalyx-preserving strategies across the kidney transplant continuum, with explicit distinction between clinical supportive principles, human observational evidence, and preclinical or mechanistic evidence.

Stage/Strategy	Mechanistic Target	Current Evidence/Level of Support	Main Translational Challenge
Donor management and ischemia minimization	Reduce endothelial priming, oxidative stress, and inflammatory activation before procurement and implantation	Clinical supportive principle; indirect human support from donor-quality, DGF, IRI, and organ-preservation literature	Effects are diffuse and difficult to isolate from broader donor-risk factors [1,2,3,4,5,11,12,19]
Hypothermic/normothermic machine perfusion optimization	Perfusion pressure, oxygenation, colloid composition, hemolysis control, endothelial exposure time	Human and experimental organ-preservation evidence; porcine NMP data indicate the glycocalyx is measurable and vulnerable during perfusion	Need graft-specific endpoints and glycocalyx-aware perfusate design [12,22]
Albumin supplementation	Supports oncotic microenvironment and endothelial barrier stability	Mainly preclinical and physiologic rationale; early urinary glycosaminoglycan reduction in a small porcine KT model	Dose, timing, and true graft-level benefit remain uncertain [10,12,30]
Sulodexide/glycosaminoglycan-oriented therapy	Potential glycocalyx rebuilding and heparanase modulation	Preclinical porcine KT proof-of-concept; supportive CKD and heparanase biology, but no human KT interventional data	Needs larger translational datasets and mechanistic confirmation in humans [21,30]
Tie2 activation/VE-PTP inhibition	Reinforces endothelial quiescence, barrier integrity, and anti-inflammatory signaling	Experimental kidney IRI and mechanistic endothelial evidence; transplant relevance remains conceptual without human KT trials	Human transplant trials are lacking; optimal timing and patient selection unresolved [33,34]
Thrombomodulin–protein C axis support	Restore anticoagulant endothelial phenotype and attenuate ischemic endothelial injury	Experimental kidney ischemia protection plus human observational sTM kinetics in early KT	Unclear whether biomarker changes translate into treatment responsiveness [23,24]
Complement modulation	Prevent complement-driven glycocalyx loss and inflammatory amplification	Experimental renal IRI evidence and strong transplant immunology rationale; glycocalyx-specific clinical evidence remains limited	Requires precise patient selection and integration with existing transplant immunology workflows [20,25]

Abbreviations: DGF, delayed graft function; KT, kidney transplantation.

## Data Availability

No new data were created or analyzed in this study. Data sharing is not applicable to this article.

## Abbreviations

The following abbreviations are used in this manuscript:

AKI	acute kidney injury
ATP	adenosine triphosphate
CKD	chronic kidney disease
DGF	delayed graft function
EG	endothelial glycocalyx
eGFR	estimated glomerular filtration rate
IRI	ischemia–reperfusion injury
KT	kidney transplantation
MMP	matrix metalloproteinase
NMP	normothermic machine perfusion
PBR	perfused boundary region
sTM	soluble thrombomodulin
Tie2	tyrosine kinase with immunoglobulin-like and EGF-like domains 2
TIMP	tissue inhibitor of metalloproteinases
VE-PTP	vascular endothelial protein tyrosine phosphatase
VEGF	vascular endothelial growth factor
ABMR	antibody-mediated rejection
EV	extracellular vesicle
MHC	major histocompatibility complex
miR	microRNA
TCMR	T-cell-mediated rejection
